# The role of the affective temperament in the treatment adherence in psychiatry

**DOI:** 10.11604/pamj.2016.25.2.8400

**Published:** 2016-09-02

**Authors:** Lilia Bahrini, Rahma Damak, Mejda Cheour

**Affiliations:** 1 Psychiatry E Department, Razi Hospital, Cité les Oranger, 2010 Manouba, Medicine University of Tunis, Tunisia

**Keywords:** Adherence, MARS adherence, affective temperaments, temperament TEMPS

## Abstract

**Introduction:**

Adherence to psychotropic medications is affected by factors related to the treatment, to the physician, to the environment and to the patient himself. The purpose of the present study was to investigate the influence of affective temperaments on treatment adherence.

**Methods:**

Thirty six stabilized outpatients were recruited from the aftercare consultation of Psychiatry to perform Temperament Evaluation of Memphis, Pisa, Paris and San Diego Auto questionnaire version (TEMPS-A) for affective temperaments and the Medication Adherence Rating Scale (MARS) for treatment adherence.

**Results:**

The total MARS score was negatively correlated with the irritable temperament score. The MARS’s score relative to the attitude of patients to psychotropic medications and their negative side effects was negatively correlated with the cyclothymic, the irritable and anxious temperaments. Patients having a diagnosis of psychotic disorder had a significantly greater medication adherence and behaviour toward medication score compared to those having a diagnosis of affective disorder. A greater MARS’s score for the negative side effects and attitudes to psychotropic medication was associated with medication by neuroleptics with prolonged action.

**Conclusion:**

The results of the present study suggest that patients with irritable temperament may have more difficult to follow psychotropic medications, and that patients with cyclothymic, irritable and anxious temperaments may be more attentive and sensitive toward psychotropic medications and their negative side effects.

## Introduction

Treatment adherence is one of the most important prognostic factors for mental illness. Haynes defines adherence as the “match between the patient's behaviour and his doctor's recommendations for a program therapy (medication, psychotherapy, hygiene of life, additional tests or presence to appointments)” [1]. The World Health Organization defines adherence as «the extent to which a person’ s behaviour-taking medication, following a diet, or making healthy lifestyle changes-corresponds with agreed-upon recommendations from a health-care provider » [2]. In general, across all medical specialties, it is estimated that 25% of inpatients and 50% of outpatients don’t take medication correctly [1, 3]. The poor adherence to treatment in psychiatry is frequent. It affects 15 to 25% of inpatients and 50% of outpatients. Twenty per cent of patients in psychiatry does not buy their drugs in the months following the prescription and 30 to 50% of medicines purchased would be not consumed [4]. In psychiatry, treatment adherence is of extreme importance, because the consequences of poor compliance are major. The consequences in terms of efficacy and safety must be firstly considered. The economic consequences of the poor compliance must also be taken into account. They are expressed in terms of direct and indirect costs associated with relapses, rehospitalisation, chronicity, or down productivity [5–11]. The determinants of adherence can be divided into four factors: factors related to treatment [9], factors related to the physician and physician-patient relationship [3, 12], factors related to the environment [9] and factors related to the patient himself. The first three factors are dynamic and it is here that most of the studies have been done [10]. The factors related to the patient are in their majority static, and an action at this level is often the most difficult. Patient’s characteristics taken into play in respect of adherence are multiple; they are both cognitive, behavioural, emotional and psychopathological. To these are added the notions of knowledge and beliefs [13–15]. Thus, many studies have highlighted that poor compliance is favoured by extreme ages, male gender, low socioeconomic level, low cultural level and psychosocial isolation and certain personalities like hypochondriac or paranoid. Meanwhile, the temperament has been little studied as a factor related to the patient and being decisive in adherence [16]. Temperaments are imperturbable variations of personality, traits and ways of reacting to the environment that characterize individuals and remain constant throughout several different situations [17]. The purpose of this study was to highlight the role of patient's affective temperament in adherence to treatment in psychiatry.

## Methods

### Participants

This was cross-sectional study. Thirty-six stabilized outpatients were recruited from the aftercare consultation of Psychiatry E department of the Razi Hospital in Tunis, over a period of three months from January to March 2015. All patients meeting the following criteria were included: literate patients and stabilized patients on treatment for more than 3 months. Were excluded all patients with a psychiatric examination showing symptoms of relapse. According to Diagnostic and Statistical Manual of Mental Disorders-Fourth Edition (Text Revision) (DSM-IV-TR), psychiatric diagnoses were schizophrenia (n=19), bipolar disorders (n=11), schizoaffective disorder (n=3) and major depressive disorder (n=3). Each participant gave his oral consent to participate in this study. The research ethic board of Razi hospital approved the study.

### Temperament assessment

The temperament was assessed using the « Temperament Evaluation of Memphis, Paris and San Diego- Auto-questionnaire » version (TEMPS-A). The TEMPS-A is based on interview versions of the depressive, cyclothymic, irritable and hyperthymic temperaments [18–22] which have been validated in an Italian population of 1010 students aged from 14 to 25 [23, 24]. The Italian study upheld the four-factor structure of « Temperament Evaluation of Memphis and Paris Interview or Italian version (TEMP-I). The present self-rated (auto questionnaire) version of TEMPS-A [25] has been enriched with the addition of an anxious temperament [26]. The TEMPS-A was translated in more than 32 languages and is used worldwide. The validated Arabic version of TEMPS-A questionnaire was used [27, 28] and proved to be suitable to the Tunisian population [29].

### Adherence measurement

The adherence was assessed using the « Medication Adherence Rating Scale » (MARS). MARS was created by Thompson and al. [30] for the assessment of adherence in psychiatric patients. They identified several deficiencies in the « Drug Attitude Inventory » (DAI) as a measure of adherence and proposed a new inventory, the MARS scale, that incorporates features of both the DAI [31, 32] and the « Medication Adherence Questionnaire » (MAQ) [33] but which they claimed to have greater validity and clinical utility. The scale includes 10 items and was first validated in patients with schizophrenia. MARS examines adherence behaviours and attitudes toward medication with relatively simplistic scoring [30]. MARS is useful in psychiatric practices or psychiatric clinic settings [30]. The MARS includes 10 yes or no items. It explores 3 factors, factor 1 is relative to the four first items that explore “the medication adherence and behaviour”, factor 2 is relative to the items 5-8 that explore ‘the attitude to taking medication’ and ‘the beliefs about medications’ and factor 3 is relative to items 9 and 10 that explore “the negative side-effects and attitudes to psychotropic medication”. The patient should be asked to respond to the statements in the questionnaire by circling the answer which best describes their behaviour or attitude towards their medication during the past week. Responses for a good adherence are “no” for the items 1-6 and 9-10 and “yes” to the items 7-8. [34–36]. Scores =5 were considered as high which mean that treatment adherence was sufficiently good and scores < 5 were considered as low which mean that treatment adherence was bad. In this study, we used the Arab version validated in Tunisian dialect. This validation was made in the context of a doctoral thesis in medicine at the Tunis Faculty of Medicine and is actually the subject of a paper being submitted.

### Statistical analysis

Data for all cases were compiled electronically and analysed using SPSS version 18. The Kolmogorov-Smirnov test was used to test the normal distribution of the data. T-test and Chi-square were respectively used for comparison of continuous or parametric variables (Mann–Whitney and Fisher exact test when appropriated). Pearson's correlation coefficients were obtained between the age, age of onset, duration of illness, number of medication, total score of MARS, MARS’s factor 1, 2 and 3 scores and temperament scores. We referred to levels of significance of p<0.05.

## Results

### Descriptive study

Socio-demographic features: the population mean age was 37 years ± 8.21 (men= 66.7% and women= 33.3%). Twenty six patients (72.2%) were single, eight of them (22.2%) were married and two patients (5.6%) were divorced. More than the half of our population (58.3%) reached the secondary education level, 27.8% the primary level and 13.9% the high school level. The family support was good in 63.9% of cases and bad in 33.3% of cases.

**Figure 1:**
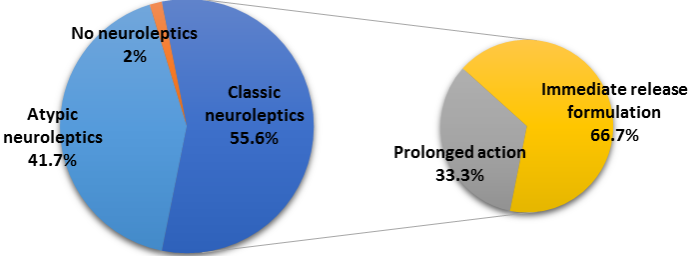
Distribution of patients by type of prescribed neuroleptic

Clinical features: sixty-one point one percent of the patients had psychotic disorders and 38.9% had affective disorders. The mean age of onset was 25.47 years ± 6.05 and the mean duration of illness was 11 years ± 8.76. Twenty three patients (63.9%) were current smoker, eleven of them (30.6%) had history of recent alcohol consumption and two had history of recent cannabis abuse. The mean number of re-hospitalizations was 3.58 ± 2.06 with a range of 1-9. Prescription rate varied between different types of psychotropic medications. The mean number of medication was 2.56 ± 0.87 with a range of 1 to 4. The Figure 1 shows the rates of the different prescribed neuroleptics. Mood stabilisers were prescribed for 44.4% of the patients.

TEMPS-A assessment: according to the TEMPS-A, anxious temperament was the more frequent temperament with a mean score of 12. 32 ± 6.25, flowed by the hyperthymic temperament (mean score 11.3±3.97), cyclothymic temperament (mean score: 11.22±5.13) and depressive temperament (mean score 11±4.09). The mean score of the irritable temperament was 7.19 ± 4.62. MARS measurement: the majority of patients; 77.8% (n=28) had a high MARS score and only 22.2% (n=8) had a low MARS score. As shown in Table 1, the mean score of the total MARS was 6.28 ± 2.37, which mean that treatment adherence was sufficiently good.

**Table 1:** Mean scores of MARS*

	Mean Score	Standard deviation	Range
**Total MARS score**	6.28	2.374	2-10
**MARS's factor 1 score**	2.56	1.34	0-4
**MARS's factor 2 score**	2.83	1.2	1-4
**MARS's factor 3 score**	1	0.828	0-2

* Medication Adherence Rating Scale

### Association between MARS scores and TEMPS-A

The results of this study showed a negative correlation between the total MARS score and the irritable temperament score (p=0.026, r= -0.37) but no correlations with the other temperaments scores. As shown in Table 2, the mean MARS’s factor 3 score was negatively correlated with cyclothymic (p=0.004; r=-0.46), irritable (p=0.004; r=-0.46) and anxious temperaments (p=0.04; r=--0.37). As shown in Table 3, the depressive and irritable temperaments were significantly associated with low MARS (p=0.022; p= 0.009).

**Table 2:** Correlations between MARS* and TEMPS-A** scores

	Total MARS score		MARS’s factor 1 score		MARS’s factor 2 score		MARS’s factor 3 score	
	p	r	p	r	p	r	p	r
**Depressive temperament**	0.068	-0.317	0.141	-0.250	0.342	-0.16	0.065	-0.31
**Cyclothymic temperament**	0.114	-0.260	0.309	-0.179	0.568	0.98	**0.004**	-0.46
**Hyperthymic temperament**	0.725	-0.06	0.71	0.64	0.966	-0.00	0.368	-0.15
**Irritable temperament**	**0.006**	-0.447	0.81	-0.294	0.505	-0.11	**0.004**	-0.46
**Anxious temperament**	0.226	-0.207	0.3	-0.178	0.316	0.172	**0.04**	-0.37

* Medication Adherence Rating Scale

** Temperament Evaluation of Memphis, Paris and San Diego- Auto-questionnaire

**Table 3:** Associations between TEMPS*-A scores and high/low MARS** score

	Patients with a high MARS score	Patients with a low MARS score	p
Depressive temperament	10.18	13.88	**0.022**
Cyclothymic temperament	10.39	14.13	0.069
Hyperthymic temperament	11.5	10.75	0.644
Irritable temperament	6.14	10.88	**0.009**
Anxious temperament	11.32	15.75	0.077

* Temperament Evaluation of Memphis, Paris and San Diego- Auto-questionnaire

** Medication Adherence Rating Scale

### Association between MARS scores and socio-demographic and clinic features

The results of this study did not show any association between socio-demographic features and the mean scores of MARS, MARS’s factor 1and 3 and the high score of MARS. The attitude to taking medication and the beliefs about medication score was significantly associated with the marital status with greater score among the married patients compared to the single and divorced ones (p=0.049). Concerning the clinical features, patients having a diagnosis of psychotic disorder had a significantly greater medication adherence and behaviour toward medication score compared to those having a diagnosis of affective disorder (2.95 ±1.32 vs 1.93 ± 1.14; p=0.023). The number of patients having a high score of MARS were significantly higher among those having a diagnosis of psychotic disorders (p=0.018). A greater score for the negative side effects and attitudes to psychotropic medication was associated with medication by neuroleptics with prolonged action (p=0.004). The patients having a high score of MARS were significantly associated with a higher mean number of re-hospitalizations (3.93 vs 2.38; p=0.05).

## Discussion

In our study, although the majority of patients had high MARS scores (77.8% having scores > 5) the mean MARS score remains low (6.28/10) indicating that patients did not have a sufficiently good adherence. Furthermore, the mean MARS’s factor 1 score was 2.56/4. This indicates that the patients’ adherence behaviour was not sufficiently good toward their medication, with frequent behaviours such as omission, oversight or discontinuation of treatment. This result is consistent with the data of literature showing that poor adherence to treatment in psychiatry is frequent [4]. The adherence to treatment remains difficult to estimate [37–39]. Several evaluation methods were proposed, they are divided into direct and indirect measurements. Direct measurements, which include measures such as detection of the drug in biological fluid [40] and direct observation of the patient taking medications, are objective methods. More commonly used are the indirect measurements including medication Event Monitoring System (pill counts) [10], self-report measures, prescription claims data and the use of adherence scales [9]. Although several studies demonstrated the superiority of the direct measures in the evaluation of the adherence to treatment, we opted in this study, for an indirect measure by using the auto-questionnaire of MARS. Although the MARS scale is subjective, it is specific to the psychiatric population. Furthermore, we have a validated version in Arabic adapted to the Tunisian dialect. The results of our study show that when taking into consideration the total score of MARS, the treatment adherence was good in 77.8% of patients. However, by considering each factor separately, the results show that the patients had a passable attitude toward taking medication and that the subjective perceptions on drugs and their negative side-effects was not good enough. Hogan and al [32] believe that the adherence to treatment is strongly linked to the unconscious effect of an aversive interoceptive conditioning. A negative perception could influence the future behaviour of the subject without his knowledge. They emphasize the subjective experience to the detriment of the only adverse effects. The way in which the patient feels his treatment, the beliefs or knowledge on the beneficial aspects, the undesirable effects and the action of the drugs influence less significantly the adherence to treatment.

### Association between adherence to treatment and the affective temperament

The results of this study showed that the depressive and irritable temperament was significantly correlated with a poor adherence to treatment. These results are consistent with those found in the study of Kamei and al [16] who assessed the adherence using the« Visual Analogue Scales » (VAS) and the « Drug Attitude Inventry-10 » (DAI-10). In fact, their results showed that VAS scores for total compliance were significantly and negatively associated with depressive temperament scores while VAS scores for compliance with dose were significantly and negatively associated with depressive temperament scores and irritable temperament scores. They also found that the VAS scores for compliance with dose and DAI-10scores were significantly and positively associated with hyperthymic temperament scores indicating that hyperthymic patients had a better adherence to treatment. In our study, the hyperthymic temperament wan not associated to a better adherence. This difference with the results of the study of Kamei and al [16], may be explained by the different tools of adherence assessment. MARS, VAS and DAI-10 can measure different aspects of adherence leading to no significant association. In our study, the cyclothymic, irritable or anxious temperaments were significantly associated with a bad « attitude to psychotropic medication and their negative side-effects scores ».

### Association between adherence to treatment and socio-demographic and clinical features

Concerning the socio-demographic features, our study has shown that married patients had a better attitude toward drugs. Kampman and al [41] showed in their study that being single is a risk factor for a poor adherence. More than being single, the fact of living alone increases the bad adherence behaviour [41–44]. The association between marital status and treatment adherence could be explained by the fact that married patients are supervised and assisted by their spouses in their daily lives. Moreover, in our study the family support has not been associated with a better adherence to treatment. Thus, the spouses seem to be better guarantors for the drug therapy than the rest of the family. Parents are often older and siblings are engaged in their own family and professional life. Our study has not found significant association between the adherence to treatment and the addictive behaviours such as smoking, alcoholism or substance use. This is inconsistent with the data from the literature [9]. Concerning the clinical features, our study has shown that patients with psychotic disorders had a better adherence and a better adherence behaviour toward medication than those with affective disorders. At first sight, this result might seem paradoxical. However, several studies have demonstrated that although the insight during the relapses is low both in schizophrenic and bipolar patients [45, 46], and that it is best among the bipolar during intervals of stabilization, the differences between the two groups are in the dimensions of the insight [47]. Indeed, after treatment the bipolar patients improve their insight of illness and of illness’s symptoms while schizophrenics improve their insight of the necessity of treatment while the insight of illness remain poor [47]. This improvement of the insight of the necessity of treatment may be explained by the fact that the schizophrenic patients have essentially positive psychotic symptoms which are known to be predictive of the improvement of insight [48]. The results of the current study showed that the score of the patient’s attitude toward psychotropic medication and their negative side effects was greater among patients treated by prolonged neuroleptic action. In fact, Samalin and al [49] found that this form of neuroleptic is still poorly used in routine. It should not be only reserved to noncompliant patients but even to those having a sufficiently good adherence to treatment to ensure the persistence of this good attitude toward medication. Patients having a higher number of hospitalizations had a higher MARS scores. This result was expected. Poor compliance is often related with a higher number of hospitalization [47].

## Conclusion

The main results of this study suggest that patients with depressive and irritable temperaments had a poor adherence to treatment. These patients require more education concerning the necessity of medication. Moreover, patients with cyclothymic, irritable and anxious temperaments are more sensitive toward the negative side effects of psychotropic medication and require more assistance and advices concerning psychotropic medication. Therefore, consideration of patients’ temperament could be improved the education which will be more targeted.

### What is known about this topic

- The non-treatment adherence is one of the main reasons for relapse of psychiatric patients;
- The determinants of treatment adherence are related to the treatment, the environment, the therapeutic alliance and certain factors related to the patient himself;
- Affective temperament plays an important role and influences human behavior.

### What this study adds

- The present study showed the influence of affective temperament on the quality of patient compliance in psychiatry;
- In the present study, depressive and irritable temperaments were associated with a poor therapeutic compliance.
